# Small RNA Profiling of Cucurbit Yellow Stunting Disorder Virus from Susceptible and Tolerant Squash (*Cucurbita pepo*) Lines

**DOI:** 10.3390/v15030788

**Published:** 2023-03-19

**Authors:** Saritha Raman Kavalappara, Sudeep Bag, Alex Luckew, Cecilia E. McGregor

**Affiliations:** 1 Department of Plant Pathology, University of Georgia, Tifton, GA 31793, USA; 2 Department of Horticulture, University of Georgia, Athens, GA 30602, USA

**Keywords:** cucurbit yellow stunting disorder virus, virus-derived small RNA, *Cucurbita pepo*, tolerance, crinivirus

## Abstract

RNA silencing is a crucial mechanism of the antiviral immunity system in plants. Small RNAs guide Argonaut proteins to target viral RNA or DNA, preventing virus accumulation. Small RNA profiles in *Cucurbita pepo* line PI 420328 with tolerance to cucurbit yellow stunting disorder virus (CYSDV) were compared with those in Gold Star, a susceptible cultivar. The lower CYSDV symptom severity in PI 420328 correlated with lower virus titers and fewer sRNAs derived from CYSDV (vsRNA) compared to Gold Star. Elevated levels of 21- and 22-nucleotide (nt) size class vsRNAs were observed in PI 420328, indicating more robust and efficient RNA silencing in PI 420328. The distribution of vsRNA hotspots along the CYSDV genome was similar in both PI 420328 and Gold Star. However, the 3’ UTRs, CPm, and p26 were targeted at a higher frequency in PI 420328.

## 1. Introduction

Cucurbit yellow stunting disorder virus (CYSDV), which causes leaf yellowing in cucurbits, is production constrained worldwide. Since its first discovery in the early 1990s in the United Arab Emirates [1], the virus has spread to regions in Asia, Europe, North Africa, and North America [2,3] over the last two decades. In the United States, CYSDV has emerged as a significant concern in the Southwest and the Southeast when the largest incidence of whiteflies coincides with the growing season [3,4,5]. CYSDV belongs to the genus *Crinivirus* in the family *Closteroviridae* [6,7] and is transmitted in a semipersistent manner by the sweet potato whitefly, *Bemisia tabaci* Gennadius [6,8]. Symptoms of the disease start with the appearance of interveinal chlorotic spots on the older leaves, which eventually fuse, leading to severe chlorosis. CYSDV can reduce plant vigor [8], decreasing yield [6,9,10,11]. 

The genome of CYSDV consists of two molecules of single-stranded (ss) RNA of positive (+) polarity denoted as RNAs 1 and 2 [12]. RNA1 is 9123 nucleotides (nt) long and encodes proteins associated with replication. The first two overlapping open reading frames (ORFs) of RNA1 possess five conserved domains, papain-like protease, methyltransferase, RNA helicase, and RNA-dependent RNA polymerase (RdRp). In addition, there is a small 5 kDa hydrophobic protein, and 2 ORFs that potentially encode proteins of 25 and 22 kDa, respectively, with no homologs in any databases. RNA2 is 7976 nt long and contains the hallmark gene array of the family *Closteroviridae*. These ORFs include a heat shock protein 70 (HSP70) homologue, a 59 kDa protein, the major coat protein (CP), and a divergent copy of the minor coat protein (CPm). The 1177 nt long 5′ non-coding region (NCR) in RNA2 is another unique feature of CYSDV as the longest NCR described among plant ssRNA viruses [12]. Most of the isolates of CYSDV, including those from Europe (Spain), the Middle East (Jordan, Lebanon, and Turkey), and North America, share more than 99% nucleotide sequence identity, while those from Saudi Arabia form a divergent group with nucleotide identity of about 90% with the remaining isolates [7]. 

As in the case of many viral diseases, CYSDV management relies heavily on the avoidance of disease by application of chemical insecticides to control whitefly vectors. However, insecticidal sprays are ineffective and unsustainable when the vector population is too high. In addition, these chemicals are hazardous to the environment and the health of humans and animals. In this scenario, host-plant resistance to the viruses or their vectors is the most effective and ecologically safe alternative for managing viral diseases. Presently, there are no commercial *Cucurbita pepo* cultivars resistant to CYSDV [13]. Identifying resistant/tolerant sources and elucidating the underlying mechanisms are essential steps toward the development of CYSDV-resistant and horticulturally acceptable varieties. 

Ongoing efforts to develop resistant cucurbit germplasm have identified host plant resistance to CYSDV in melon (*Cucumis melo*) accessions PI 313970 [14] and TGR-1551 (PI 482420; subsp. *melo*) [10]. *C. pepo* PI 420328 was identified to potentially have tolerance to CYSDV in multi-location trials conducted under open-field conditions [15]. This work further confirms previous findings of tolerance in *C. pepo* PI 420328 to CYSDV. The potential mechanism underlying the tolerant phenotype in PI 420328 is not known.

RNA silencing, also known as RNA interference (RNAi), is a conserved biological response to double-stranded RNA (dsRNA) that regulates gene expression and is considered the primary antiviral defense mechanism in plants [16]. There are several key reactions and proteins involved in this process. The first step is activated by dsRNA formed either as replication intermediates of viruses, imperfect RNA hairpins formed at some areas of the viral genome, or by overlapping sense–antisense transcripts produced by circular ssDNA genomes as in the case of geminiviruses [17]. Second is the processing of the dsRNA into small RNAs (sRNAs), and it is catalyzed by isoforms of the RNase-III-type Dicer-like protein (DCL) [17]. In the third step, small interfering RNAs (siRNAs) derived from the pathogen’s genomes are loaded into effector complexes called RNA-induced silencing complexes (RISCs), having a member of the Argonaute (Ago) protein family at its core. The sRNAs act as sequence-specific determinants that guide RISCs to complementary RNA or DNA. Antiviral silencing is achieved by either post-transcriptional gene silencing (PTGS) mediated by 21 or 22 nt sRNAs, where the target is completely cleaved, or by transcriptional silencing of viral DNA, mediated by 23 nt sRNAs [16]. 

Here, we report a comparative study of viral sRNA profiles in CYSDV susceptible and tolerant genotypes of *C. pepo*.

## 2. Materials and Methods

### 2.1. Plant Materials and CYSDV Culture

Squash (*Cucurbita pepo* L.) PI 420328, shown to have field tolerance to whitefly-transmitted viruses [15], was obtained from the USDA-Agricultural Research Service germplasm collection. Squash cultivar Gold Star, susceptible to CYSDV [18], was sourced from Seedway, LLC (Hall, New York, NY, USA). The isolate of CYSDV used in this study was collected in the fall of 2020 from squash grown in a University of Georgia (UGA) research plot in Tifton, GA, USA. In the phylogenetic analysis published earlier, the CYSDV isolates from Georgia (MW685460, MW685459 and MW685457, MW685458) were shown to share more than 99% nucleotide sequence identity with other isolates of CYSDV reported from the USA and Europe [4]. Since CYSDV is not mechanically transmitted [8], the CYSDV culture was maintained on squash variety Gold Star by whitefly transmissions as described earlier [19]. Whitefly populations used for inoculations were reared on cotton (DG3615, B3XF), a non-host of CYSDV [20]. 

### 2.2. Virus Inoculation 

**Figure 1:**
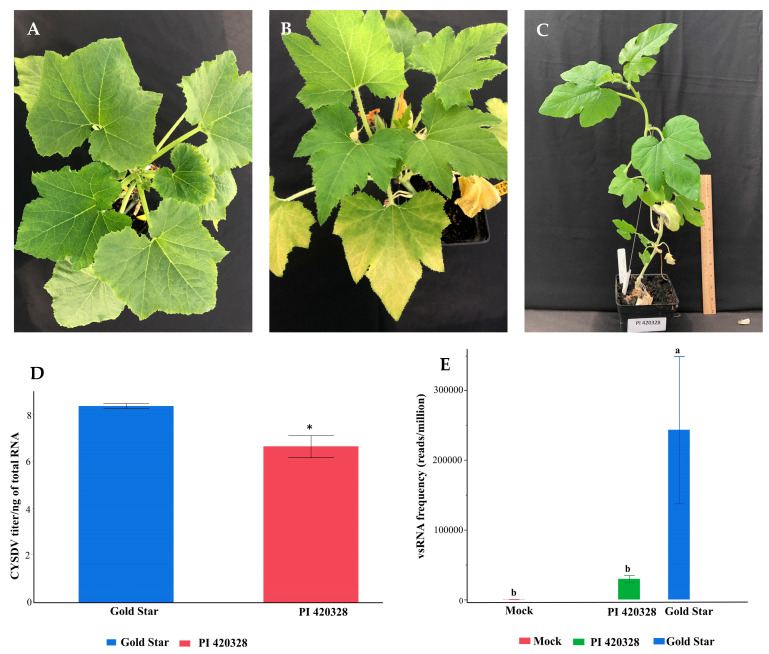
Impact of CYSDV infection on squash line Gold Star and PI 420328, 30 days after inoculation: (**A**): mock-inoculated plants with non-viruliferous whiteflies displayed no phenotypic symptoms; (**B**): Gold Star developed prominent interveinal chlorosis and yellowing; (**C**) PI 420328 only displayed yellowing symptoms on the lower leaves; (**D**) estimated copy numbers of CYSDV in Gold Star (blue) and PI 420328 (red) at 30 DAI. Viral titer is represented as the mean Log concentration per ng total RNA. Each value is the average of three biological replicates with standard error bars; * indicates a significant difference (*p* < 0.05); (**E**) number of CYSDV-derived vsRNA reads per million total reads averaged over three replicates. Levels not connected by the same letter are significantly different (Student’s *t*-test, *p* < 0.05).

Whiteflies reared on cotton were released on squash leaves infected with CYSDV for 48 h for virus acquisition [Acquisition access period (AAP)]. As with other members of the genus *Crinivirus*, transmission of CYSDV occurs in a semipersistent manner [6]. Following AAP, whiteflies were aspirated, and a group of 30–50 whiteflies were allowed to feed on the abaxial surface of the test squash plants using clip cages for 48 h for inoculation [Inoculation access period, (IAP)]. Mock inoculations were performed by allowing 30–50 whiteflies collected directly from cotton to feed on Gold Star. All inoculations were performed on two-week-old squash seedlings with two true leaves. Clip cages were removed 48 h post-inoculation, and the whiteflies were killed by treating recipient plants with neonicotinoid insecticide ASSAIL^®^ 30SG (UPL NA Inc. King of Prussia, PA, USA) containing the active ingredient acetamiprid at a rate of 0.025 g (a.i.)/100 mL water. This ensured the symptoms of CYSDV would not be confused with squash silverleaf disorder, a condition caused by persistent feeding by immature whiteflies [20,21]. Inoculated plants and controls were maintained inside the cages (BugDorm, 160 µm aperture, MegaView Science Co., Ltd. Taichung, Taiwan) in the greenhouse facility at the University of Georgia, Tifton. The greenhouse was maintained at a temperature of 28 ± 3 °C and 50 ± 20% relative humidity throughout the experiment. The third true leaf from the inoculated plants were collected 30 days after inoculation (DAI) (Figure 1) and frozen immediately in liquid nitrogen for sequencing and virus quantification.

### 2.3. RNA Isolation and Small RNA Sequencing

A total of nine samples were prepared for sequencing: three biological replicates each of tolerant (PI 420328), susceptible (Gold Star), and mock (Gold Star) inoculated squash. Approximately 100 mg of frozen tissue was ground in liquid nitrogen, and the total RNA was extracted using Spectrum™ Plant Total RNA Kit (Sigma Aldrich, St. Louis, MO, USA), following manufacturer’s instructions. The integrity and purity of the total RNA was determined using a Qubit and quantified using a NanoDrop-2000 spectrophotometer (Thermo Scientific, Wilmington, DE, USA). RNA samples with OD_260/280_ in the range of 1.8–2.2, OD_260/230_ ≥ 2.0 and RIN value ≥8.0 were used to construct the cDNA library. Small RNA libraries were constructed and sequenced on the Illumina platform (single-end read 1 × 50 bp; Novogene, Sacramento, CA, USA). 

### 2.4. Quantification of CYSDV 

RT-qPCR assays in a quantitative system using SSOAdvanced Universal SYBR Green Supermix (Bio-Rad, Hercules, CA, USA) were performed to quantify CYSDV. The reactions were performed in a CFX96 Touch Deep Well Real-Time PCR System (Bio-Rad, Hercules, CA, USA). Primers and PCR profiles published earlier for CYSDV quantification were used in qRT-PCR [22]. Each biological sample was tested in triplicate. cDNA was prepared from 100 ng of total RNA using CYSDV RdRp-specific primers [22] following the protocol described earlier [23]. An amount of 5 µL of cDNA was used as a template in the qPCR reaction of 25 µL, which also consisted of 12.5 µL of SSOAdvanced Universal SYBR Green Supermix and 1 µL each (10 µM) of forward and reverse primers. An initial denaturation step (3 min at 95 °C) was followed by 40 cycles of denaturation (10 s at 95 °C) and a combined step of annealing and extension at 62 °C for 30 s. Melting curve analysis was performed to evaluate the specificity of the fluorescence signal. The cycle thresholds and melting curve were calculated by CFX Maestro Software (Bio-Rad, USA). 

Virus titers were estimated following protocols described earlier [18,19]. Cycle threshold (Ct) values from samples were compared with an external standard to estimate the copy number of CYSDV present in a sample. The standard consisted of a series of six ten-fold dilutions of plasmid containing the fragment of the CYSDV RdRp gene. RNA concentration of the plasmid was measured in ng/μL using a Nanodrop (Thermo Scientific, Wilmington, DE, USA), and the number of copies was estimated based on the formula: the number of copies = (amount in ng * 6.022 × 10^23^)/(length of a vector in bp * 1 × 10^9^ * 650), in which the weight of a base pair (bp) is assumed to be 650 Da [24]. Three technical replicates of one microliter from each dilution was used as the template in qRT-PCR.

### 2.5. Analysis of sRNA Sequences

Removal of low-quality sequences and adapters was performed with CLC Genomics Workbench 21 (Qiagen, Redwood City, CA, USA). Only reads within the size range of 20–25 nt were retained. To retrieve and count CYSDV-derived small RNAs, clean sRNA reads were aligned to the reference sequence of CYSDV (RNA 1; NC_004809.1 and RNA 2; NC_004810.1), allowing zero mismatches. The number of each size class of virus-derived small RNA (vsRNA) was also determined using CLC for quantitative comparisons. The distribution of vsRNAs along the virus genome and the 5′ terminal nucleotides of the small RNAs was determined using MISIS-2 [25,26]. The number of reads that map to different regions was determined from the single nucleotide resolution map of 20–25 nt vsRNAs derived from CYSDV generated by MISIS-2 (Supplementary Tables).

### 2.6. Statistical Analyses

Data analyses were performed in JMP^®^, Version 16 (SAS Institute Inc., Cary, NC, USA, 1989–2021). Before analysis, the data for CYSDV titer were log-transformed, and the number of vsRNA in each size class was converted to a percentage. The vsRNA aligning to the UTRs and cistrons were normalized as frequency/100bp of gene/million reads. Differences in CYSDV titer, percentage of each nt size class, and frequency of vsRNA aligning to different regions between Gold Star and PI 420328 were analyzed by *t*-test. Treatment means were considered significant at *p* < 0.05. The number of CYSDV-derived reads in Gold Star, PI 420328, and mock were analyzed using one-way ANOVA. Treatment means were separated with a post hoc *t*-test. 

## 3. Results

### 3.1. Symptom Severity and Virus Titer Is Higher in Susceptible (Gold Star) Than Tolerant (PI 420328) C. pepo 

Following whitefly-mediated transmission of CYSDV, Gold Star and PI 420328 plants were monitored for symptom expression. Interveinal chlorosis was observed as early as two weeks post-inoculation on the lower leaves of susceptible cultivar Gold Star. The symptoms progressed rapidly, with interveinal chlorosis becoming more severe and spreading onto the upper leaves. On the other hand, plants from PI 420328 remained asymptomatic until three weeks post-inoculation. Delayed symptoms of mild interveinal chlorosis were observed on the lower leaves at 21 days after inoculation (DAI). The symptoms remained mild, and only the bottom leaves displayed prominent yellowing after 30 DAI when samples were collected for sequencing. The mock-inoculated (non-viruliferous whiteflies) plants of Gold Star did not exhibit any phenotypic symptoms of interveinal chlorosis or yellowing (Figure 1A–C). The estimated copy numbers of CYSDV in PI 420328 were significantly lower compared to susceptible Gold Star (*t*-test, *p*= 0.03; Figure 1D).

### 3.2. Elevated Levels of 22 and 21 nt Size Classes in Tolerant (PI 420328) C. pepo

Total sRNA populations from mock-inoculated, as well as susceptible and tolerant lines of *C. pepo*, were sequenced to compare the interaction of CYSDV with small(s) RNA silencing pathways during susceptible and tolerant interactions. Read statistics from the nine small RNA libraries generated from CYSDV-infected Gold Star, PI 420328, and mock-inoculates are presented in Supplementary Table S1. To determine the amount of vsRNA in each library, clean sRNA reads were aligned to the reference sequence of CYSDV (NC_004809.1 and NC_004810), allowing zero mismatches. The vsRNA reads were negligible in the mock-inoculated plants. However, these were subtracted while determining total vsRNA reads from Gold Star and PI 420328. Overall, the number of reads aligning to the CYSDV genome from three biological replicates of PI 420328 were significantly lower than in Gold Star (Anova, Students *t*-test, *p* = 0.04) and comparable to those in mock (Anova, Students *t*-test, *p = 0.03*) (Figure 1E). 

**Figure 2:**
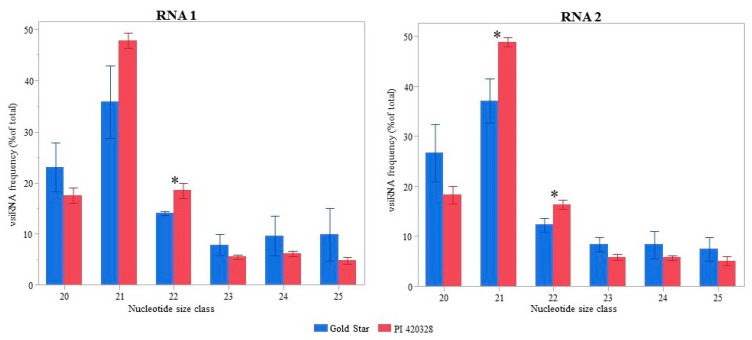
Relative abundance of different size classes in total vsRNAs 30 days after inoculation in susceptible (Gold Star) and tolerant (PI 420328) squash, systemically infected with cucurbit yellow stunting disorder virus (CYSDV). Each value is the average of three replicates with standard error bars. Asterix ^(^*^)^ indicates that corresponding values in Gold Star and PI 420328 are significantly different (*t*-test, *p* < 0.05).

The proportion of different size classes of vsRNA in Gold Star and PI 420328 were compared. The most predominant size class obtained post-infection was the 21 nt followed by the 22 nt size class in both Gold Star and PI 420328 (Figure 2). However, the percentage of 22 nt (*p* = 0.03) and 21 nt (*p* = 0.04) aligning to the CYSDV genome was significantly higher in PI 420328 (Figure 2). The percentage of 21 nt aligning to the CYSDV genome was 35.8% in Gold Star while in PI 420328 21 nt vsiRNA comprised 47.7% of total vsiRNA. The 22 nt vsiRNAs comprised 13.9% in Gold Star, while in PI 420328 it was 18.4%. The frequency of vsRNAs of less abundant size classes (20, 23, 24, and 25) were higher in Gold Star than in PI 420328 (Figure 2). 

### 3.3. Origin of Viral siRNAs

**Figure 3:**
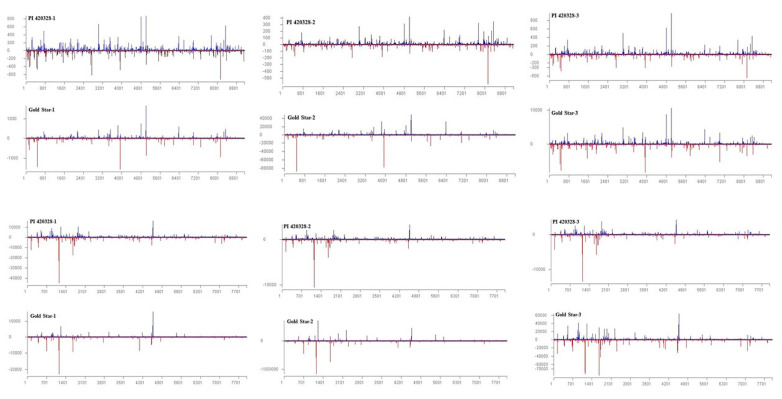
Distribution of vsRNAs along the genomes of CYSDV in PI 420328 and Gold Star 30 days after inoculation with cucurbit yellow stunting disorder virus (CYSDV). The histograms plot the numbers of 21 to 24 nt viral siRNA reads at each nucleotide position of CYSDV genomic RNA 1 and RNA 2, (mapped with zero mismatches). The bars above the axis (blue) represent sense (forward) reads starting at each position, and those below (red) represent antisense (reverse) reads ending at the respective position.

**Figure 4:**
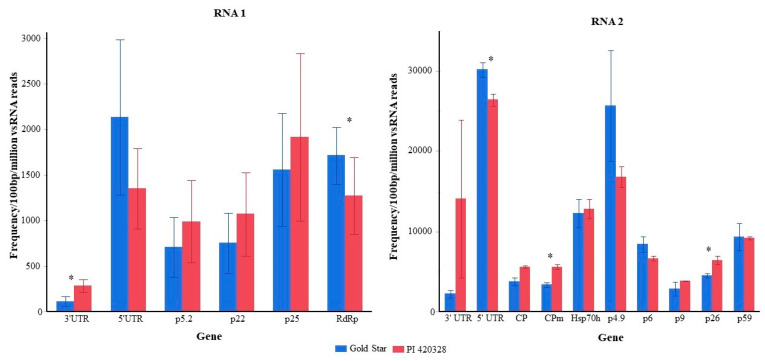
Accumulation of 21–25 nt virus-derived sRNAs corresponding to UTRs and cistrons of CYSDV in *Cucurbita pepo* leaves at 30 DAI. Values are the average of three biological replicates and are represented as the frequency of vsRNA/100 bp of gene length/million total vsRNA reads. Asterix ^(^*^)^ indicates that corresponding values in Gold Star and PI 420328 are significantly different (*t*-test, *p* < 0.05).

Virus-derived sRNAs originated from the entire length of RNA1 and RNA2 of the CYSDV genome and in both polarities in both PI 420328 and Gold Star (Figure 3). The pattern/location of hotspot distribution along the genome of the virus was similar in CYSDV-infected PI 420328 and Gold Star (Figure 3). To ascertain the differences in frequency of vsRNAs targeting distinct regions of CYSDV in Gold Star and PI 420328, vsRNAs were mapped to individual cistrons of CYSDV as well as the 5′ and the 3′ untranslated regions (UTR). The abundance of vsRNAs was normalized to the length of the cistron (reads/100 bp of the gene) and total vsRNA reads (reads/million total reads) generated in the library (Figure 4). 

The 5′ UTRs of RNA 1 and RNA 2 were the most targeted region of CYSDV genome in both PI 420328 and Gold Star. This was followed by p4.9 located on RNA2 and p25 and RdRp located on RNA1. Some regions were targeted more frequently in PI 420328 than in Gold Star. These included the 3′ UTR of RNA 1 (*p* = 0.02) as well as CPm (*p* = 0.005) and p26 (*p* = 0.04) located on RNA 2. In contrast, the vsRNA reads targeting RdRp and 5′ UTR of RNA 2 (*p* = 0.03) were significantly higher in Gold Star than in PI 420328 (*p* = 0.03) (Figure 4). 

### 3.4. 5′-Terminal Nucleotide Distribution

**Figure 5:**
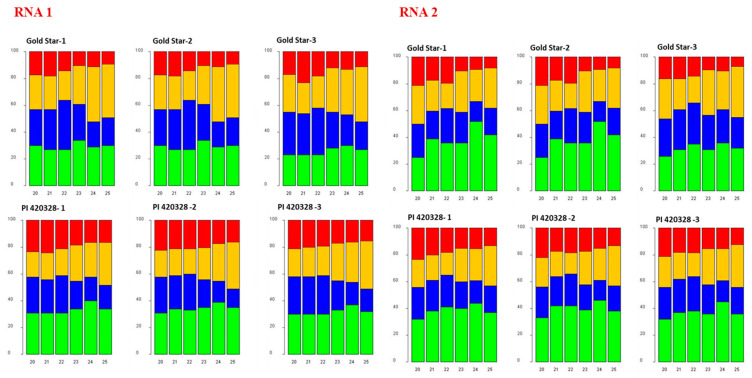
The 5′-terminal nucleotide frequency of 20–24 nt CYSDV-derived small RNAs (sRNA) in Gold Star and PI 420328. The composite bar graphs indicate the percentage of 5′ U (red), 5′ G (orange), 5′ C (blue), and 5′ A (green) for each of the size classes of virus CYSDV-derived sRNAs.

The predominant 5′ nucleotide was 5′ A on both 21 nt and 22 nt vsRNA from both RNAs of CYSDV in all replicates of PI 420328 (Figure 5). The percentage of 21 nt vsRNA with 5′ A varied from 30 to 34% and 37 to 43% on vsRNA derived from RNA1 and RNA2, respectively. The percentage of 22 nt vsRNA with 5′ A varied from 30 to 33% on RNA1 and 38 to 41% on RNA2. The percentage of vsRNA with 5′ A was lower in all replicates of Gold Star than in PI 420328. In Gold Star, the percentage of 21 nt vsRNA with 5′ A varied from 23 to 26% and 23 to 38% on 5′ RNA 1 and RNA2 respectively. The percentage of 22 nt vsRNA ranged from 22 to 27% on RNA1 and 34 to 35% on RNA2. The percentage of vsRNA with 5′ U did not show any trend, as displayed by 5′ A in PI 420328 and Gold Star. It was also observed that 5′ C was the most abundant 5′ terminal nucleotide in 22 nt RNA1 of Gold Star. 

## 4. Discussion

Tolerance has been defined as a mitigation of the impact of virus infection irrespective of the pathogen load [27]. Even though PI 420328 sustained a significant CYSDV load, the symptoms were milder compared to those in susceptible squash. RNA silencing is a well-established antiviral immunity system in plants [16,17,28]. To investigate whether sRNA responses are associated with tolerance to CYSDV in PI 420328, the vsRNA population was characterized and compared between PI 420328 and Gold Star at 30 DAI. The number of CYSDV-derived sRNAs was lower in PI 420328 than in Gold Star (Figure 1E), similar to the trend in the estimated copy number of the virus in both these lines. This indicates a more efficient silencing mechanism of viral RNA in PI 420328, leading to a lower concentration of the virus and less severe symptoms. The margin of difference in estimated copy number was lower than vsRNA reads. One reason could be the use of RdRp gene-directed primers to estimate copy numbers. A significantly higher number of vsRNA targeted this gene in Gold Star than in PI 420328. Similar results were observed earlier in the case of cassava infected with Ugandan cassava brown streak virus [29] and in tomatoes infected with TYLCV [30], where resistant genotypes produced fewer amounts of vsRNAs in comparison with the susceptible ones. The size distribution of vsRNAs in a plant is determined by the type of DCL acting [31]. DCL2 [32], DCL3 [33], and DCL4 process long dsRNA molecules into siRNA populations that are 22, 24, and 21 nt in length [33,34,35,36].

Among the size classes important in RNAi in both Gold Star and PI 420328, 21 nt followed by 22 nt were prominent. Interestingly, however, the percentages of 21 nt and 22 nt size classes in total vsRNA were higher in PI 420328 compared to those in Gold Star for RNA2 (Figure 2). The elevated levels of 22 nt in tolerant line is interesting in light of their prominent role in antiviral RNA silencing. A growing body of evidence demonstrates the advantages of having a higher percentage of 22 nt vsRNA in establishing a higher level of resistance to viruses [37]. The first evidence relates to the hierarchical functioning of DCL1, DCL4, and DCL2. Accordingly, when viral RNA levels are low early in an infection cycle, DCL1 and DCL4 would dominate RNA silencing and produce limited amounts of 21 nt sRNAs. However, as the level of viral RNA increases, DCL1 and DCL4 would be saturated, and DCL2 begins to act, making 22 nt viral sRNAs [28,34]. The threshold of sRNA concentration at which DCL2 begins to act could be lower in the case of PI 420328 and lead to the production of 22 nt vsRNA much earlier than in Gold Star, thus launching a more robust RNA silencing response against CYSDV and maintaining a virus titer lower than Gold Star. 

More and more studies have demonstrated that DCL2-generated 22 nt sRNAs can trigger transitive PTGS. DCL2 promotes secondary sRNA production more efficiently, thus adding to the amplification property of the RNA silencing pathway in an infected plant. This is achieved by the 3′ ends of 22 nt sRNA that protrude from the sRNA–AGO nucleoprotein and bind to the protein SGS3. This AGO–SGS3 complex stalls ribosome translocation, thus repressing viral mRNA translation. The protruding 3′ end of the 22 nt sRNA is then available as a primer for more viral dsRNA production by host RDR [37]. The dsRNAs are the substrates DCL4 uses to increase the quantity of 21 nt secondary vsiRNAs. The newly synthesized dsRNAs lead to the synthesis of multiple secondary sRNAs. Finally, DCL2 plays an important role in systemic silencing, whereby RNA silencing from the initially infected cells moves with or ahead of the virus and influences its accumulation elsewhere in the plant [38,39]. Some evidence indicates that 22 nt sRNAs produced by DCL2 are strongly associated with mobile sRNA silencing, for example, between host plants and *Cuscuta campestris* parasitic plants [40]. There is also evidence that DCL2-processed 22 nt L-siRNA partially comprises the bona fide signals for induction of non-autonomous PTGS in *N. benthamiana* [41].

The vsiRNA hotspots are regions of the viral genome that are highly targeted during RNAi [42,43,44,45]. However, the viral sRNA biogenesis of CYSDV has not been investigated so far. Analysis of single nucleotide-resolution maps revealed that viral siRNAs are spawned along the entire length of RNA1 and RNA2 of CYSDV with local hotspots evident on the forward (viral sense) and reverse (viral complementary) strands in both genotypes (Figure 3). In addition, the pattern of distribution of vsiRNA and location of hotspots were similar in Gold Star and PI 420328. Viral siRNA biogenesis is often governed by differences in viral genome sequences and independent of the host [43,46,47] with few exceptions [44,48]. The hotspot patterns are distinct for each virus and could be used to develop pathogen-derived resistance by transgenics, expressing viral dsRNA optimal for RNAi or designing RNA optimal for exogenous application to activate RNAi machinery [49].

The number of vsiRNA targeting each gene co-related with the size of the genes in both Gold Star and PI 420328 (Supplementary Figure S1). However, there was a difference in the frequency of vsRNA targeting different genes in PI 420328 and Gold Star (Figure 4). In particular, the frequency of vsRNA targeting the 3′ UTR of RNA 1, 5′ UTR of RNA 2, as well as CPm and p26 located on RNA2 was significantly higher in PI 420328 compared to Gold Star. Interestingly, CPm has been found to have an RNA silencing suppressor role in another *Crinivirus*, ToCV [50].

The sorting of small RNAs into Arabidopsis Argonaute complexes is directed by the 5’ terminal nucleotide [51]. To determine potential interactions between vsiRNAs with distinct AGO complexes, we analyzed the relative abundance of 5′-terminal nucleotides of vsiRNAs. For the predominant class of 21 and 22 nt vsiRNAs, A was the most abundant nucleotide at the 5′-end, while U was the least abundant. This indicates that vsRNAs of 21 and 22 nt size classes derived from CYSDV are preferentially loaded into AGO2 and/or AGO4, due to their preference for A at the 5′-termini [52]. AGO1 and AGO2 are the two major plant antiviral Argonautes that function against RNA viruses [53]. The 21 and 22 nt sRNAs with 5′ U are predominantly loaded onto AGO1 and those with 5′ A onto AGO2. A nucleotide bias toward adenine (A) or uracil (U) over cytosine (C) and guanidine (G) at the 5′ terminal end is typical of vsiRNAs in various organisms, including plants, fungi, and insects [44,51,54,55].

The predominance of 21 or 22 nt vsRNA during plant virus infection is well-established [32,56,57]. However, to the best of our knowledge, there is only one other published example where a resistant genotype changes the siRNA profile of a plant virus. Similar results of elevated levels of 22 nt were observed in resistant tomato encoding *Ty1* gene upon infection by tomato yellow leaf curl virus [58]. Considering the recent findings highlighting the role of DCL2-mediated RNA silencing pathways [28,37,38,41,59] over other DCLs in antiviral silencing, the higher proportion of 22 nt and in turn 21 nt vsRNAs is indicative of a more stringent PTGS pathway in PI 420328 compared to Gold Star. 

In this study, the higher abundance of 21 and 22 nt vsiRNAs in PI 420328 and a higher percentage of 21 and 22 nt svRNAs with 5′ A could indicate the role of PTGS in rendering it tolerant to infection from CYSDV. PI 420328 will be a potential source of tolerance to CYSDV in breeding programs. Future research will aim to identify the host loci associated with tolerance and introgressing these loci into summer squash. 

## Data Availability

Not applicable.

## Supplementary Materials

The following supporting information can be downloaded at: https://www.mdpi.com/article/10.3390/v15030788/s1, Figure S1: Accumulation of 21 to 25 nt virus-derived sRNAs per cistron in CYSDV in inoculated *Cucurbita pepo* leaves. Values are the average and standard error of three biological replicates and are represented as a percentage of total virus-derived sRNAs. Genes are arranged in the order of their size on the x-axis. Each value is the average of three replicates with standard error bars. Table S1: Read statistics from the nine small RNA libraries generated from CYSDV-infected Gold Star, PI 420328, and mock-inoculates.
