# Supplementary material for: Small RNA Profiling of Cucurbit Yellow Stunting Disorder Virus from Susceptible and Tolerant Squash (Cucurbita pepo) Lines
Source: Viruses. 2023 Mar 19;15(3):788. doi: 10.3390/v15030788 (PMC10058471; doi:10.3390/v15030788)
Supplement: Supplementary file 1 [file viruses-15-00788-s001.zip › viruses-2269863-supplementary.pdf]

Supplementary Figure

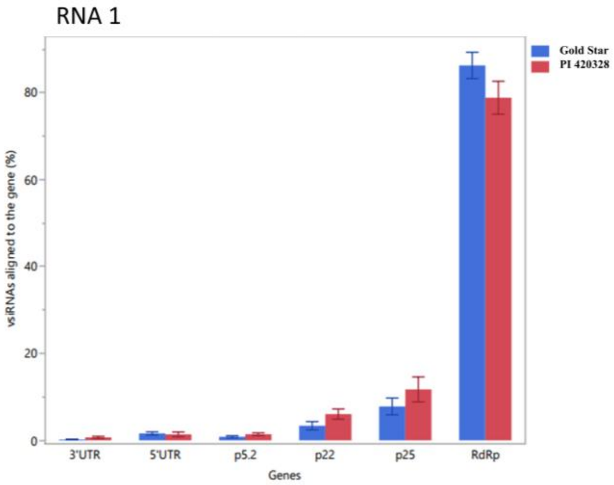

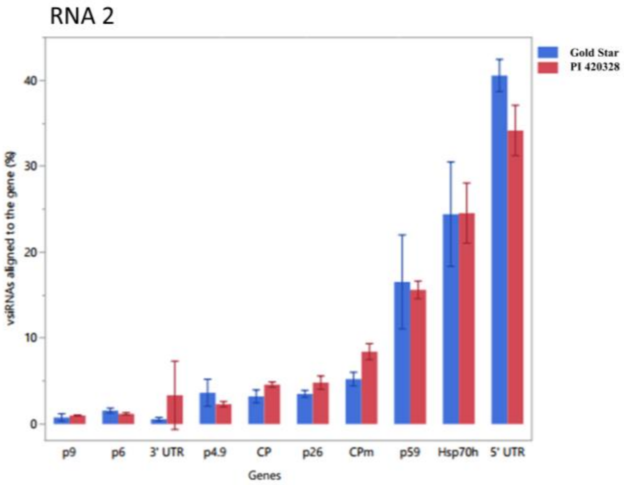

Figure S1. Accumulation of 21 to 25 nt virus-derived sRNAs per cistron in CYSDV in inoculated *Cucurbita pepo* leaves. Values are the average and standard error of three biological replicates and are represented as a percentage of total virus-derived sRNAs. Genes are arranged in order of their size on the x-axis. Each value is the average of three replicates with standard error bars.

Table S1: Read statistics from the nine small RNA libraries generated from CYSDV-infected Gold Star, PI 420328, and mock-inoculates.

**Samples**	**Total reads**	**Adapter trimmed reads**	**20-24nt reads**	**Total reads aligned to CYSDV**
Mock-1	26,281,657	26,245,609	4,024,031	1596
Mock-2	23,239,317	23,211,374	7,354,968	1712
Mock-3	26,152,695	26,121,232	16,092,407	4572
PI 420328-1	22,033,282	22,016,803	15,484,162	517129
PI 420328-2	25,314,986	25,291,176	18,791,162	641889
PI 420328-3	23,754,847	23,735,568	15,787,762	961098
Gold Star-1	23,904,217	23,820,879	6,744,415	928516
Gold Star-2	110,334,924	110,246,449	56,288,932	43223842
Gold Star-3	31,709,425	31,666,639	17,501,754	9492950
